# Enhanced Photoluminescence of R6G Dyes from Metal Decorated Silicon Nanowires Fabricated through Metal Assisted Chemical Etching

**DOI:** 10.3390/ma16041386

**Published:** 2023-02-07

**Authors:** Ioannis Kochylas, Anastasios Dimitriou, Maria-Athina Apostolaki, Maria-Christina Skoulikidou, Vlassios Likodimos, Spiros Gardelis, Nikolaos Papanikolaou

**Affiliations:** 1 Section of Condensed Matter Physics, Department of Physics, National and Kapodistrian University of Athens, Panepistimiopolis, 15784 Athens, Greece; 2 Institute of Nanoscience and Nanotechnology, NCSR “Demokritos”, Aghia Paraskevi, 15310 Athens, Greece

**Keywords:** surface enhanced photoluminescence, silver nanostructures, gold nanoparticles, Rhodamine 6G, metal-assisted chemical etching

## Abstract

In this study, we developed active substrates consisting of Ag-decorated silicon nanowires on a Si substrate using a single-step Metal Assisted Chemical Etching (MACE) process, and evaluated their performance in the identification of low concentrations of Rhodamine 6G using surface-enhanced photoluminescence spectroscopy. Different structures with Ag-aggregates as well as Ag-dendrites were fabricated and studied depending on the etching parameters. Moreover, the addition of Au nanoparticles by simple drop-casting on the MACE-treated surfaces can enhance the photoluminescence significantly, and the structures have shown a Limit of Detection of Rhodamine 6G down to $10^{-12}$ M for the case of the Ag-dendrites enriched with Au nanoparticles.

## 1. Introduction

The optical properties of metallic nanostructures based on noble materials such as Ag, Au, Pt, and Pd have been studied extensively. In particular, their ability to concentrate light to volumes with subwavelength dimensions led to several proposals for their use in optical sensors [1,2,3,4,5]. Among noble metals, silver has attracted a lot of attention as it is a rather inexpensive metal with good optical properties thanks to its narrow plasmon resonance inside the visible spectrum, leading to the use of silver substrates in various applications such as biosensing [6,7], imaging, drug delivery [8], solar cells [9,10], and catalysis [11]. These active substrates can amplify light due to the excitation of coherent electron oscillations, also known as Surface Plasmon Polaritons (SPP), and enhance the electromagnetic field near the surface of metal nanostructures [12,13,14]. These effects were used in various surface-enhanced phenomena such as Surface Enhanced Raman Spectroscopy (SERS) [15,16] or Surface Enhanced Photoluminescence Spectroscopy [17] (SEPS), and Surface Enhanced Fluorescence (SEF) [18,19]. In particular, structures such as silver nanoshells [20], nanocubes [21,22], nanoplates [23] nanoclusters [24,25], dendrites [26,27,28], nanorods, nanowires [29,30] and aggregates [31,32], can offer high Electromagnetic field (EM) Enhancement Factors (EF). However, it is important to note that the size, shape, and morphology of the structures have a significant effect on the optical properties and influence the wavelength and width of the SPP resonance band of the metal nanostructures [13,15,33]. Various dye molecules such as Rhodamine 6G (R6G) [34], and Crystal Violet on rough surfaces have been demonstrated to experience significant enhancement in the optical absorption, and emission spectra [35].

Metallic nanoparticles in solution or deposited on surfaces are most commonly fabricated with chemical methods. Silver nanoparticles can be produced by the reduction of ${\mathrm{AgNO}}_{3}$ to colloidal Ag in the presence of a reducing agent [36]. Substrates prepared by the deposition of Ag or Au on nanostructured, pre-patterned surfaces, either lithographically or using templates [15,37,38] to produce ordered metallic arrays, are also considered in an effort to improve uniformity over the sensor surface. Many reviews exist in the literature [1,38,39,40] covering different aspects.

Bimetallic Au-Ag core-shell nanostructures and dendrites [27,41,42,43,44,45,46,47,48,49] have also attracted some attention since they have shown improved SEF and SERS compared to single metal Au or Ag structures [40,41,46]. The position of the plasmon resonance was controlled through the alloy composition to custom-design substrates that match the Raman excitation or the dye emission band. Generally, the plasmon resonance red-shifts with increasing Au concentration in Ag-Au alloys, but careful control of the composition is required to avoid spectral broadening and resonance damping [49].

Dendritic nanostructures have shown potential as SERS chemosensors. In particular, fractal 3D Ag-dendrites were reported for the SERS detection of lysozyme, attesting an enhancement factor of $2.4\times 10^{6}$ [50]. A similar stable, reusable, and sensitive SERS substrate was developed on a Si wafer by using the method of hydrothermal corrosion to form the Ag nanostructures, followed by a chemical reduction to add an Au nanolayer shell on the surface of the Ag dendrites. The resulting substrate could detect R6G molecules with concentration as low as $10^{-8}$ M [47]. Similar dendritic Au-Ag structures were fabricated on Cu wires and showed high detection sensitivity ($10^{-13}$ mol/L) for R6G and the antibacterial agent ciprofloxacin ($10^{-9}$ mol/L) [51]. Recently, we have demonstrated Ag-aggregates, and dendritic structures produced by MACE on Si nanowires [16] that showed high enhancement factors up to $10^{10}$ for R6G detection. In comparison, the Ag-aggregates substrates could detect R6G concentrations down to $10^{-13}$ M using SERS.

While most studies so far focus on sensors using SERS (see, e.g., reference [16] and references therein), which can be highly specific and allows for multianalyte detection, the present work aims at fluorescence detection, which is simpler and more cost-effective. However, reliable SEF-based sensors should be sensitive and precise, while multianalyte detection is possible using different emission wavelengths [18]. In this work, we concentrate on the SEF effect on R6G dyes on 3D Ag-aggregates and Ag-dendrites on 3D Si nanowire substrates. Moreover, we consider the influence of Au nanoparticles (AuNPs) after simple drop-casting on the nanostructured Ag substrates. Since many biosensor protocols are developed using AuNPs [40] the combination with Ag-nanostructured substrates could lead to improved sensors.

## 2. Materials and Methods

### 2.1. Materials

In our approach, we used Silicon Nanowires (SiNWs) as templates to grow silver nanostructures by Metal Assisted Chemical Etching (MACE) procedure [52], which was developed initially for Metal-Insulator-Semiconductor capacitors and solar cells in our group [53,54]. The initial idea was to increase the reactive surface area by the three-dimensional morphology. We explored the performance of two different types of silver nanostructures: dendrites and aggregates.

**Figure 1:**
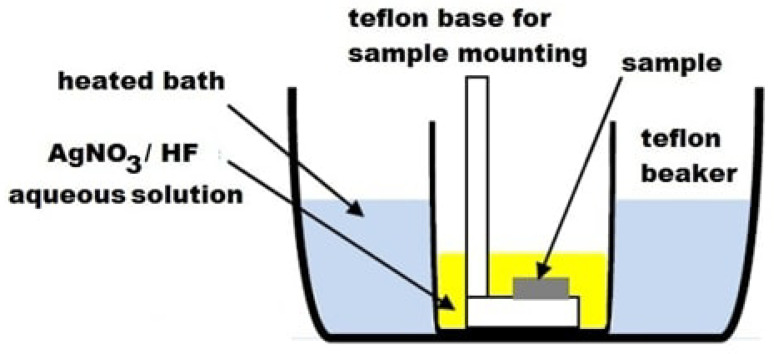
Schematic of the setup used for sample fabrication using the single-step MACE technique.

A p-type (100) oriented monocrystalline Si wafer with a resistivity of 6–8 Ω·cm was etched by immersion into a solution containing 1 g ${\mathrm{AgNO}}_{3}$, 52 mL hydrofluoric acid (HF) and 273 mL deionized (D.I.) water (solution pH = 1). Figure 1 presents a schematic of the experimental setup used for the MACE process. The temperature of the solution was kept constant at 35 °C for the whole etching process. MACE is an anisotropic etching process, and the SiNWs were developed vertically, in the (100) direction [52,55].

We used two different methods to develop the active substrates. In the first method, the Si wafer was immersed in the etching solution for a few minutes. This produced SiNWs and micrometer-sized Ag-dendrites at the tips and between the SiNWs. In the second method, an etching time of 6 min was chosen for producing samples with SiNWs. Subsequently, the as-formed SiNWs were dipped into an aqueous HNO_3_ solution using deionized (D.I.) water D.I./HNO_3_ 1:1 by volume in order to dissolve and remove the silver nanostructures that were grown during the MACE process. Then, the substrates with the bare SiNWs were dipped in a ${\mathrm{AgNO}}_{3}/\mathrm{HF}$ aqueous solution with the same chemical composition as in the initial step for 7 s, in order to decorate the SiNWs surface with Ag-aggregates.

We evaluated the plasmon-induced enhancement of the Photoluminescence (PL) signal of R6G. Here, we note that the SiNWs were non-luminescent. Moreover, AuNPs, of about 70 nm in diameter, were added to the Ag-decorated substrates, and their influence on the PL enhancement of the R6G analyte was studied. In particular, a drop of 40 μL Sigma-Aldrich AuNPs stabilized in 0.1 mg/mL sodium citrate buffer was cast on the different substrates and was allowed to get adsorbed and dried before the deposition of R6G.

For all experiments, a 50 μL drop of an aqueous solution of R6G of various concentrations was cast on the substrates and then was adsorbed, dried, and measured under the same conditions. Solutions with varying concentrations were obtained starting from an initial molarity of $10^{-5}$ M, which was prepared by weighing a certain amount of R6G powder and dissolving it in a certain volume of ultrapure water. By calibrated absorption measurements, we managed to obtain accurate concentrations between $10^{-6}$ M and $10^{-12}$ M by diluting the initial concentration of $10^{-5}$ M in different amounts of ultrapure water using the dilution equation: $C_{1}V_{1}=C_{2}V_{2}$ where $C_{1}$ and $C_{2}$ are the concentrations before and after the dilution whereas $V_{1}$ and $V_{2}$ are the corresponding solution volumes.

### 2.2. Methods

Diffuse reflectance measurements were obtained by a Cary 60 UV-Vis (Agilent, Santa Clara, CA, USA) spectrophotometer equipped with a fiber optic diffuse reflectance accessory (Barrelino). PL measurements were carried out in two different experimental setups. In the first one, a green diode laser (532 nm wavelength) was used to illuminate the sample, at a 45-degree angle, with the use of a focusing lens to collimate the laser beam; the illuminated spot on the sample was approximately 2 mm wide. A long pass filter blocked the excitation laser, and the PL emission was collected with a fiber normal to the surface and analyzed using a spectrophotometer (Ocean Optics, Hong Kong). The power of the laser on the sample was 8 mW. At this laser power, there was no indication of heating or quenching of the R6G photoluminescence intensity for illumination times up to 15 s. The acquisition time was kept as short as possible ranging from 0.5 s for high concentrations to 6 s for low concentrations. PL measurements were also performed on a micro-PL experimental setup where a 523 nm diode laser was focused on the substrates by a 4× objective lens which allows the illumination of a small area of the sample, approximately 40 μm in diameter. The signal was collected through the objective, filtered, and analyzed by a spectrophotometer (Ocean Optics). The laser power on the sample in this setup was approximately 6 mW.

The structural investigation was carried out by Scanning Electron Microscopy (SEM) in a JEOL/JSM 7401F Field Emission SEM. Electron Dispersive Spectroscopy (EDS) was also performed and presented in the Supplementary Section.

## 3. Results

### 3.1. Structural Characterization

**Figure 2:**
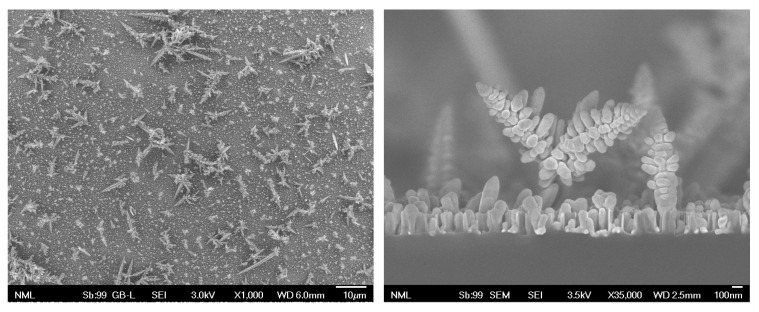
SEM micrographs of Ag-dendrites grown on SiNWs on a Si substrate, (**left**): top view (scale bar: 10 μm), (**right**): cross section (scale bar: 100 nm).

Figure 2 depicts SEM micrographs of the top-view and cross-section of the substrates produced by the first etching method, which resulted in SiNWs of height 250–300 nm and as-grown Ag-dendritic structures with heights up to several micrometers. For a 3.5 min etching time, these structures are about 2 μm high and become higher as the etching time increases. The etching time affects the morphology of the substrates as Si is etched and Ag-nanostructures grow. This is discussed in Supplementary S1, and the growth evolution can be followed in Figure S1.

**Figure 3:**
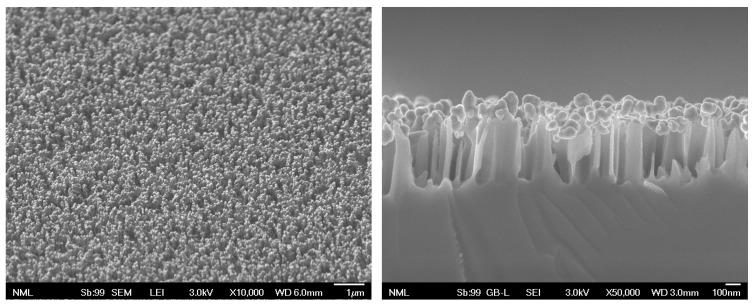
SEM micrographs of Ag-aggregate samples on the SiNWs, (**left**): top view (scale bar: 1 μm), (**right**): cross section (scale bar: 100 nm). The Ag-aggregates grow at the tips of the SiNWs.

The second etching method resulted in SiNWs of about 440–480 nm long, and Ag-aggregates, 120–150 nm in size, formed at the tips of the SiNWs as shown in Figure 3. The presence of Ag was investigated by EDS microanalysis discussed in Supplementary S2 and shown in Figure S4. The size of the Ag-aggregates increased by increasing the re-immersion time in the MACE solution. Gold nanoparticles were added by simple drop-casting, and their distribution across the surface was also investigated by EDS (Figures S3 and S5).

### 3.2. Diffuse Reflectance Measurements

**Figure 4:**
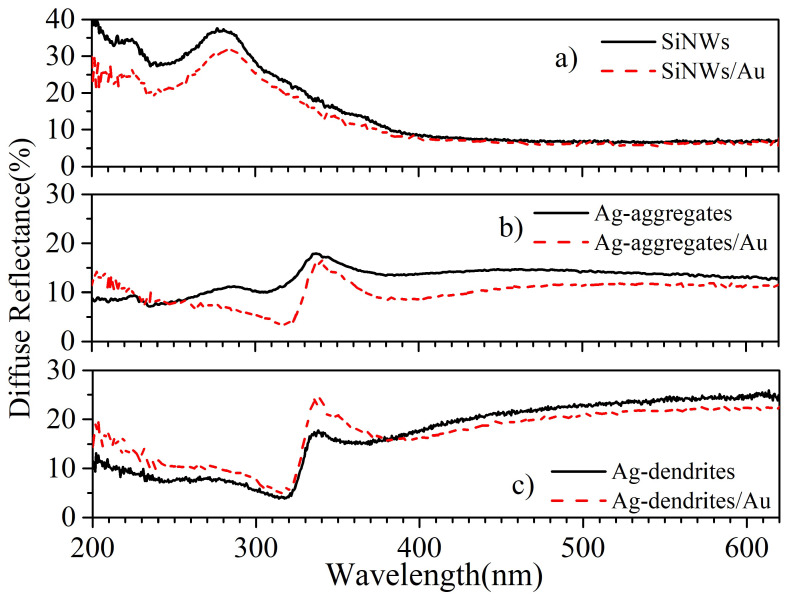
Diffuse reflectance spectra for the different substrates considered in this work: (**a**) Bare SiNWs (full line) and SiNWs enriched with 70 nm diameter AuNPs (dashed line, SiNWs/Au); (**b**) Ag-aggregates (full line) and Ag-aggregates enriched with 70 nm diameter AuNPs (dashed line, Aggregates/Au). Structures are shown in Figure 3; (**c**) Ag-dendrites (full-line) and Ag-dendrites with 70 nm diameter AuNPs (dashed line, Ag-Dendrites/Au). Structures are shown in Figure 2.

To evaluate the optical response of our proposed structures, diffuse reflectance spectra were obtained from the different substrates and compared with spectra from the same samples enriched with the AuNPs. This is shown in Figure 4a, for bare SiNWs. The peaks around 225 nm and 280 nm are expected in the reflection spectrum of bulk Si [56], while the presence of the Au reduces the reflectance slightly. The spectra for the Ag-decorated structures are shown in Figure 4b,c. Ag-dendrites are more reflective compared to the Ag-aggregates, especially for longer wavelengths. This is expected since the metal coverage is lower for the Ag-aggregates, as confirmed by the EDS analysis (Supplementary S2). The spectra of these samples show a sudden drop close to 330 nm, which is due to the abrupt change in the Ag optical constants at this wavelength [57]. A smaller drop with a minimum around 370 nm exists for the Ag-aggregates (Figure 4b), while the addition of the Au-NP results in a lower reflectivity-minimum that shifts towards slightly higher wavelengths, 380–390 nm. This can be attributed to light absorption due to particle-plasmon excitation [16] since the plasmon resonance in bimetallic Ag-Au nanostructures shifts to longer wavelengths as the Au concentration increases [42], and this was also observed in Ag-Au core-shell dendritic nanoforests [27]. The plasmon resonance shift by adding Au on the Ag nanostructures can be more clearly seen in the case of dendrites (Figure 4c) where the minimum observed around 360 nm shifts to 390 nm when Au is added.

The optical response of Ag-nanoparticles on Si wires has been addressed before [58] using 3D finite element simulations. The main conclusions were that a standalone Ag nanoparticle supports a particle mode, that can be tuned by the Ag cap size and can be hybridized by leaning of Si pillars, as well as a cavity resonance, that is controlled by the size of the Si pillar. For dendrites, the theoretical finite difference time domain simulations have shown that strong electric fields develop in the narrow gaps in the dendritic branches [47] due to plasmonic excitation, while the EM field can be enhanced if AuNPs are present.

### 3.3. Photoluminescence Measurements

**Figure 5:**
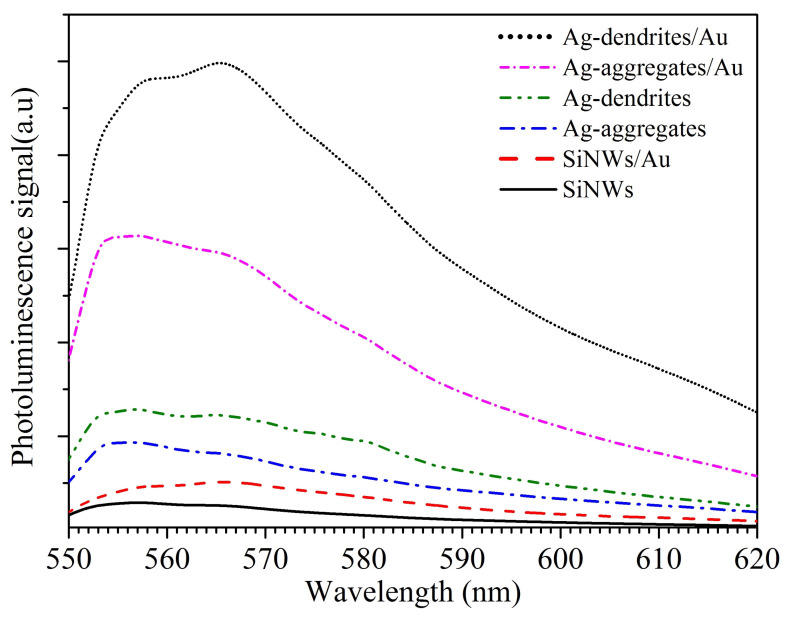
Photoluminesence spectra with a green laser excitation (532 nm) of the R6G analyte on the different substrates fabricated in this work.

Photoluminescence measurements were obtained using R6G dye as an analyte. The presence of metallic nanostructures influences the PL of the analyte through the enhancement of the EM field provided by the plasmonic excitation. Additionally, the quantum yield and the decay rate of the light-emitting dye critically depend on the distance from the metal when the analytes are found a few nm away [18,39]. Figure 5 shows the PL spectra of R6G (10${}^{-5}$ M) on bare SiNWs, Ag-aggregates, and Ag-dendrites before and after the deposition of AuNPs. The presence of AuNPs on SiNWs results in a small enhancement of PL while a maximum intensity at around 565 nm is expected for R6G. Stronger enhancement is achieved by the Ag-aggregates while Ag-dendrites increase the PL signal further. The strongest PL enhancement is observed for Au-enriched substrates. The Ag-dendrites/Au substrates are enhancing the PL signal almost 1.5 times in comparison with Ag-aggregates/Au, 5 times in comparison with Ag-aggregates and Ag-dendrites, and 20 times in comparison with bare SiNWs.

**Figure 6:**
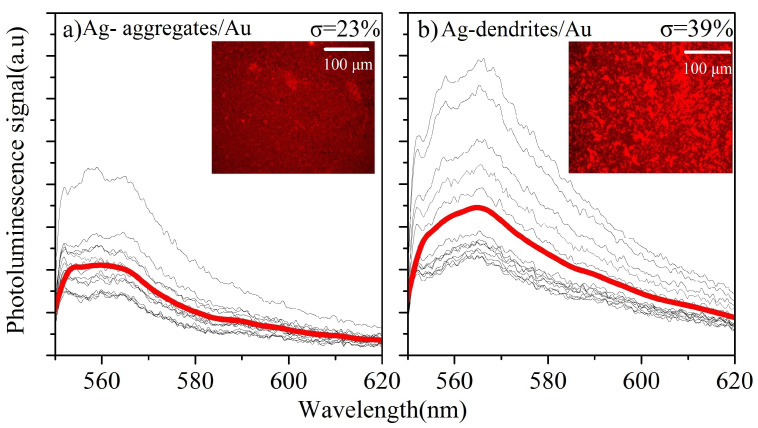
Micro-PL mapping signal comparison of (**a**) Ag-aggregates/Au and (**b**) Ag-dendrites/Au. Each plot shows N = 14 measurements at different points on the sample and the bold line represents the average signal. The relative standard deviation of the PL peak intensity (σ) was σ=23% for Ag-aggregates/Au and σ=39% for Ag-dendrites/Au. The inset shows typical fluorescence microscope images obtained with a TexasRed dichroic filter (see text).

Mapping measurements on a micro-PL setup were also carried out to investigate the homogeneity of the PL spectra across the surface of the samples. Figure 6 shows the micro-PL spectra for R6G ($10^{-5}$ M) at different locations on the Ag-aggregates/Au and Ag-dendrites/Au samples. A series of fourteen measurements were performed on areas not containing hot spots all across the sample. Typical images on a fluorescence microscope using a dichroic filter (540–575 nm excitation, 595–665 nm emission, TexasRed) are shown in the insets of Figure 6. From the spectra, it is clear that the Ag-dendrites/Au substrates show stronger PL enhancement but are less homogeneous compared to the Ag-aggregates, as is evident from the relative standard deviation.

**Figure 7:**
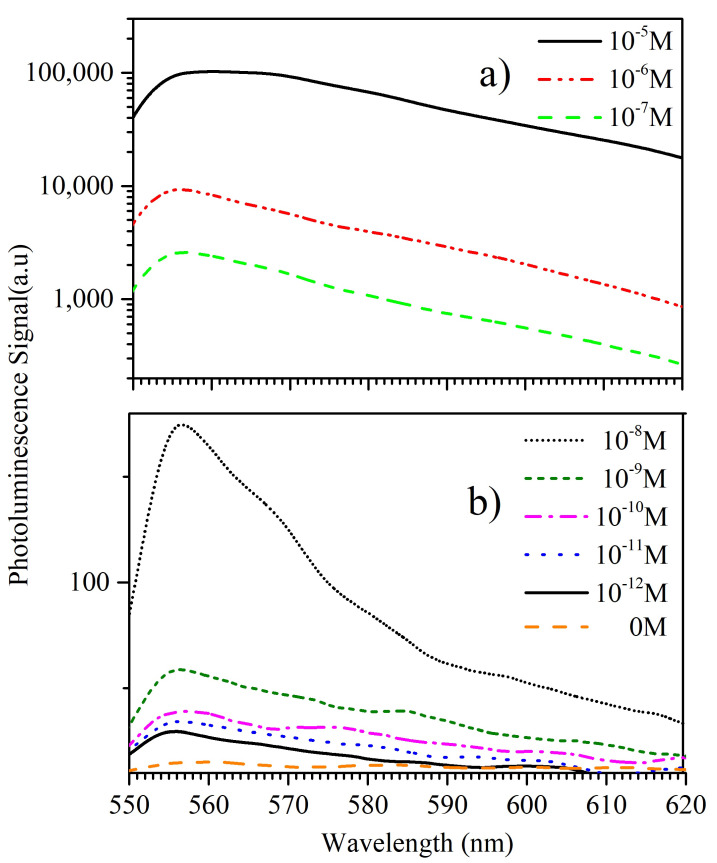
Photoluminescence spectra for various R6G concentrations (**a**) in log scale for the higher concentrations and (**b**) in linear scale for lower concentrations, obtained on Ag-dendrites/Au substrates in comparison with PL signal from R6G-free substrate.

To test the detection limit of R6G on the proposed surfaces, we vary the concentration of R6G from $10^{-5}$ M to $10^{-12}$ M for the Ag-dendrites/Au substrates. The collected PL spectra are shown in Figure 7. Each spectrum is the average of three measurements taken at different positions across the sample.

For higher R6G concentration, in order to avoid saturation of the detector, different acquisition times were used, but the spectra were re-normalized and shown on a log scale in Figure 7a. We observe a broadening of the PL spectra, while for lower concentrations shown in Figure 7b, the PL has a narrower peak around 560 nm. As seen in previously published work [59], PL emission for R6G solutions with high concentration ($10^{-3}$ M) has a peak at wavelengths above 600 nm, while for lower concentrations (≤$10^{-9}$ M) the emission maximum is expected around 550 nm. The two spectral bands observed for intermediate concentrations originated from R6G monomers and dimers. At high concentrations, the R6G molecules arrange themselves in various dimers, or larger aggregates [60]. The dimer formation has a light emission of around 600 nm. On the other hand, at low concentrations, most dye molecules are isolated from each other and fully solvated. Hence, the monomer fluorescence dominates, resulting in an emission band around 555 nm. Therefore, the broadening of the spectrum observed in Figure 7a can be the result of the combined presence of monomers and dimers, as mentioned above.

**Figure 8:**
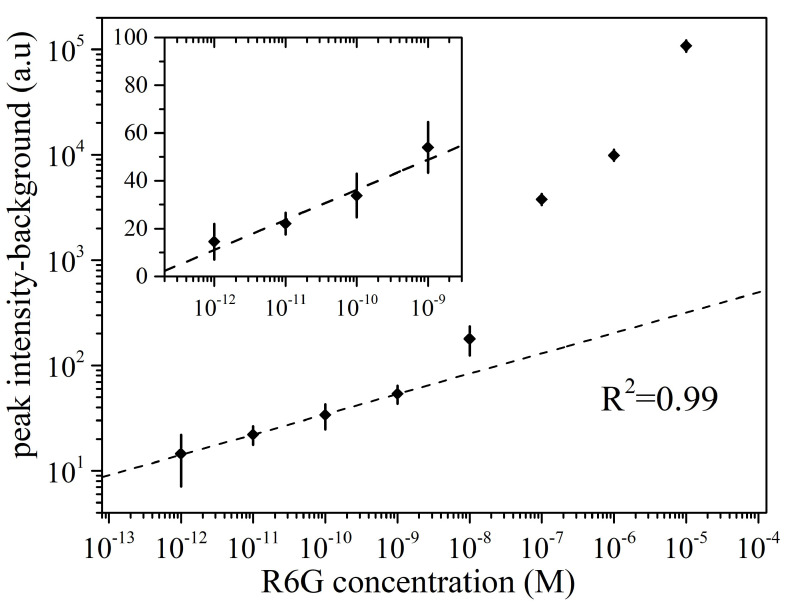
PL peak signal for various R6G concentrations (in log-log scale) for silver dendrites on SiNWs enriched with AuNPs. The plot shows the background-subtracted maximum PL signal. Analyte-free substrates are used to obtain the background signal.

In Figure 8, we present the PL peak intensity, after removing the background peak signal, for different R6G concentrations. Previous studies based on SERS of R6G show the existence of two zones, the saturation zone for high concentrations and the quantification zone for low concentrations, starting approximately below $10^{-8}$ M [48,61]. Our results show that the PL intensity for concentrations in the quantification zone can be nicely fitted in the log-log plot ($R^{2}$ = 0.99). The inset of Figure 8 depicts this low concentration region in more detail, in linear scale. As the concentration decreases below $10^{-8}$ M, only a few analyte molecules exist within the laser spot area (Supplementary Figure S5). This explains the error bars in this region. Given the error bars in the lowest concentrations, the Limit of Detection(LOD) for R6G in the case of the Ag-dendrites/Au templates is of the order of $10^{-12}$ M. It is worth noting that the LOD determined through micro-PL measurements was higher, approaching $10^{-9}$ M.

## 4. Discussion

Our results show a clear increase in the PL enhancement for Ag-dendrites compared to the Ag-aggregates. This is due to the EM enhancement in the narrow gaps of the dendritic branches. Moreover, as seen in Figure S8, even at the lower concentration, hot spots appear with the morphology of the Ag-dendrites, as discussed in Supplementary S4. The PL enhancement is more uniform for Ag-aggregates. However, fewer hot spots exist for low concentrations, so the limit of detection for R6G is worse for Ag-aggregates. The addition of AuNPs increases the PL of R6G even on the bare SiNWs substrates (without Ag) as seen in Figure 5, (black, full-line vs red, dashed-line). The PL signal increases more on Ag-aggregates and Ag-dendritic substrates compared to Au NPs on SiNWs. The EF for SEF of the R6G, calculated in Supplementary S5 is found to be $1.1\times 10^{4}$ for the Ag-aggregates/Au and $1.8\times 10^{4}$ for Ag-dendrites/Au.

The micro PL setup was used to evaluate the homogeneity and the presence and density of hot spots which appear due to sharp edges and narrow gaps that support the strong enhancement of the electromagnetic field [37]. Because of the small sampling area on the micro-PL setup, strong anisotropy due to the presence of hot spots is expected, and several measurements are required to ensure reproducibility, as can be seen in Figure 6. On the contrary, illuminating a larger area resulted in more homogeneous PL spectra which show smaller variations.

The addition of Au clearly affects the diffuse reflectance spectrum but the effect is rather small in the spectral region around 550 nm which is the emission band of R6G. We have seen no morphological change, observable through SEM, on the substrates by adding the AuNPs. As seen from the EDS (see Supplementary S2) the drop-casted AuNPs are distributed on the surface while their density is seen to vary in both the aggregates and the dendrites substrates. The etching time influences the morphology of the dendrites as is discussed in Supplementary S1, thus influencing the plasmon resonance frequency of the substrates. Optimum EM field enhancement was obtained when fine structures are developed, while the PL enhancement drops as the etching time increased. Electromagnetic simulations on the effect of AuNPs on Ag-dendritic structures reported that an Au particle diameter of 90 nm produced stronger enhancement compared with 120 nm. For larger diameters, a decrease in the EM field along the dendritic branches was predicted [51]. Moreover, a similar study on SERS enhancement of Ag-Au nanoparticles on Si nanowires [48] found strongly enhanced SERS when Ag-rich Ag-Au nanostructured alloys were formed using similar chemistry as in the present publication. The same authors reported that electromagnetic simulations showed that the particle size had a more obvious effect on the electric field than Au contents due to the low Au contents.

The increased SEF for Au NPs enriched nanostructured Ag is interesting, and similar effects for bimetallic Ag-Au alloys and core-shell nanostructures have been observed in other works [27,43]. However, the effects are reproduced in the present study, even by simple drop-casting Au NPs on an Ag nanostructured surface. We have considered the influence of AuNPs diameter on the SEF by using particles of 5 and 70 nm diameter. Our results (Supplementary S3) show that even the 5 nm nanoparticles produce a significant enhancement of the SEF on Ag-aggregates, which cannot be explained by the EM field enhancement induced by the presence of Au, similar to reference [48].

It is worth noting that the influence of charging of the metallic nanoparticles on the adsorption of Rhodamine 6G and its effect on SEF and SERS was also studied before and the difference in dependence of SERS vs SEF on the distance between the metal and the dye was identified as a key factor [62]. Therefore, the PL enhancement induced by the metallic nanostructured substrates we studied can be, most probably, attributed to a combination of EM-field enhancement due to the Ag-nanostructures and a chemical enhancement due to the presence of AuNPs. In particular, AuNPs might have a stronger affinity to R6G compared to Ag, which enhances the PL [63].

Our approach could be useful for bio and chemo-sensors based on AuNPs [40] since we have demonstrated that simple drop-casting on the Ag-nanostructured substrates could strongly enhance the PL of R6G and improve the limit of detection. However, a better understanding of the observed PL enhancement of the Au-enriched substrates is required, and the performance of the substrates using different analytes should be investigated. Further optimization of the dendrite density could also increase the uniformity and reproducibility of the SEF, especially for lower analyte concentrations.

## 5. Conclusions

In this work, we have studied the efficiency of SiNWs decorated with Ag-nanoparticles to enhance the PL intensity of the R6G analyte. The single-step MACE process was used to fabricate active substrates on Si wafers developed by two different routes. The first route led to the growth of Ag-aggregates mainly at the tips of the SiNWs, while the second route produced Ag-dendrites. Additional enrichment with AuNPs resulted in significant PL signal enhancement. The PL spectra of R6G showed great sensitivity for concentrations down to $10^{-12}$ M for Au-enriched Ag-dendrites. Our results complement previous studies that focused on biosensors based on SERS using similar structures, while our approach could be useful for improving sensor protocols based on AuNPs. However, an important aspect that deserves more thorough study in the future is the mechanism of chemical bonding of R6G to Au-Ag nanostructured surfaces.

## Data Availability

Data are available from the authors upon reasonable request.

## Supplementary Materials

The following supporting information can be downloaded at: https://www.mdpi.com/article/10.3390/ma16041386/s1. References [64,65] are cited in the Supplementary Materials.
