# Supplementary material for: Enhanced Photoluminescence of R6G Dyes from Metal Decorated Silicon Nanowires Fabricated through Metal Assisted Chemical Etching
Source: Materials (Basel). 2023 Feb 7;16(4):1386. doi: 10.3390/ma16041386 (PMC9963757; doi:10.3390/ma16041386)
Supplement: Supplementary file 1 [file materials-16-01386-s001.zip › materials-2160686-supplementary.pdf]

SUPPLEMENTARY INFORMATION

Enhanced Photoluminescence of R6G dyes from metal decorated silicon nanowires fabricated through Metal Assisted Chemical Etching

Ioannis Kochylas[1], Anastasios Dimitriou[2] , Maria-Athina Apostolaki[1], Maria-Christina Skoulikidou[2], Vlassios Likodimos[1] , Spiros Gardelis[1] and Nikolaos Papanikolaou[2]

1 Section of Condensed Matter Physics, Department of Physics, National and Kapodistrian University of Athens, Panepistimiopolis, 15784, Greece
2 Institute of Nanoscience and Nanotechnology, NCSR "Demokritos", Aghia Paraskevi, Athens, 15310, Greece

* Correspondence: n.papanikolaou@inn.demokritos.gr

S1. Scanning Electron Microscopy images for different MACE etching times.

During the MACE process several etching times were tested for the case of Ag-dendrites and on Figure S1 are displayed SEM micrographs with the produced substrates. Figure S1.a corresponds to an etching time of 2 minutes. Figure S1.b corresponds to an etching time of 3.5 minutes while Figure S1.c corresponds to an etching time of 5 minutes.

For etching times of 2 and 3.5 min we see the creation of dendritic structures on the substrates but in the first one it is evident that the size of the dendrites produced is very small. For an etching time of 5 minutes we see that we have no dendritic structure at all after the MACE process.

The supremacy of Ag-aggregates with etching time of 3.5 minutes is also evident on Figure S2 where the R6G PL signal produced is almost two times more than the one from dendrites with 2 minutes etching time.

Therefore this etching time was chosen for the fabricated substrates in this work.

S2. Scanning Electron Microscopy/ Energy Dispersive Spectroscopy (SEM/EDS).

Elemental mapping distributions of Ag, Au and Si were obtained by EDS microanalysis, at acceleration voltage of 20 kV, using an Xplore-15 SDD detector (Oxford Instruments) with a surface of 15 mm^2 and a JEOL JSM 7401F Field Emission Scanning Electron Microscope.

The results can be seen in figure S3 for the case of Ag-Aggregates/Au, figure S4 for the case of Ag-Aggregates, figure S5 Ag-dendrites/Au and figure S6 for the case of Ag-dendrites. For all cases a) represents the EDS analysis b) the SEM image c) the EDS mapping image of Ag and d) the EDS mapping image of Au.

The EDS characterizations showed that the atom percentage of Si dropped from more than 80% on the case of aggregates to ~30% for the case of dendrites. On the contrary the atom percentage of Ag increases from ~8% to ~65%. On figures S3.b and S4.b we can see that the Ag Aggregates are formed on the tips of SiNWs while figures S5.b and S6.b show that the dendrites are formed mainly from Ag while Si is located mostly on the bottom of the substrate.

As far as the Au is concerned we see that for the case of aggregates, Au has a tendency for creating clusters forming mainly larger nanoparticles on top of the Ag decorated SiNWs while in the case of dendrites, Au nanoparticles seem to be more be distributed more uniformly on top of the Ag dendrites.

S3. Dependence of the PL signal from the size of the Au nanoparticles.

On Figure S7 we display the PL Signal of R6G (10^{-5} M) on Ag-aggregates with Au nanoparticles of two different sizes (5 nm and 70 nm) in comparison with simple Ag-aggregates (without Au) and SiNWs. It is evident that Au nanoparticles with 70 nm diameter give better PL Signal enhancement and therefore they are the ones chosen for the structures fabricated in this work.

S4. Fluorescence microscope images on Ag-Dendrites/Au.

On Figure S8 we see typical images on a fluorescence microscope with 40× lens using a dichroic filter (540-575 nm excitation, 595-665 nm emission, TexasRed) from three different low R6G concentrations on Ag-dendrites/Au. The R6G concentrations displayed are 10^{-10} M (S8. a), 10^{-11} M (S8. b), 10^{-12} M (S8.c). From the three spectra it is clear that as the R6G concentration decreases so does the fluorescence signal. Especially on the lowest concentration (10^{-12} M) we see that the fluorescence signal comes mainly from hotspots while on 10^{-11} M and especially on 10^{-10} M the signal is more homogeneous.

S5. Enhancement Factor (EF).

The enhancement factor (EF) is an extremely important parameter in evaluating the Surface Enhanced Fluorescence (SEF) or Surface Enhanced Raman Spectroscopy (SERS) signal enhancement ability of different materials. However, the empirical methods for estimating EF are still controversial and not unified, although the concept of EF by SEF is clearly defined.

The AEF of an substrate strongly depends on the SEF conditions, such as morphology of substrate, excitation wavelength, etc., and can be calculated by the formula [1][2]:

$$AEF = \frac{I_{PL}/C_{PL}}{I_{NOR}/C_{NOR}}$$

where I_{PL} is the integrated SEF signal of R6G on an active substrate and I_{PL} represents the integrated normal PL signal (non-SEF) of R6G on plain Si surface. C_{PL} is the R6G concentration (10^{-5} M) in the SEF spectrum, C_{NOR} is the concentration of R6G (2.1×10^{-3} M) in normal PL.

For the Au decorated substrates in our work the AEF were: $AEF_{Ag\text{-}dendrites/Au} \approx 1.8\times10^4$ and $AEF_{Ag\text{-}aggregates/Au} \approx 1.1\times10^4$.

SUPPLEMENTARY INFORMATION

Supporting Figures:

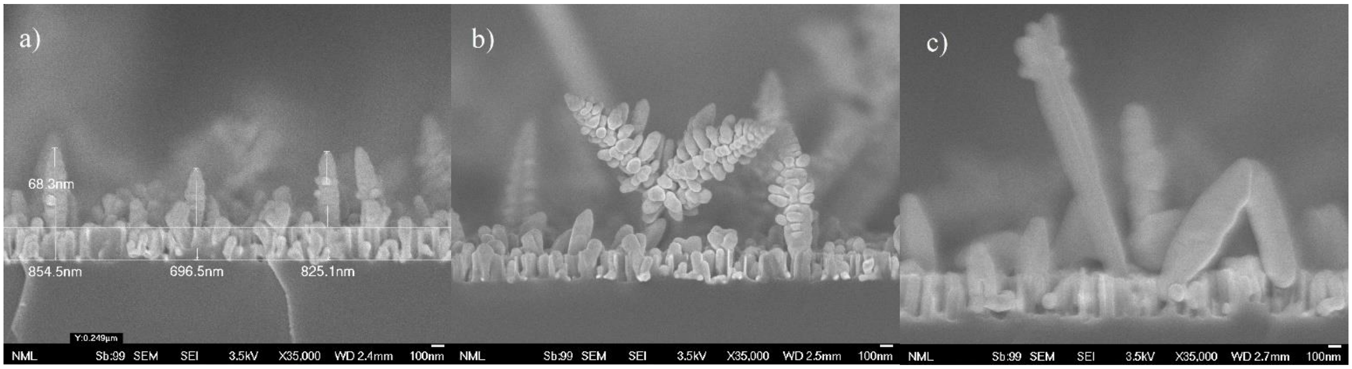

Figure S1. SEM images for Ag-dendrites with different etching times during the MACE process. a) 2 min b) 3.5 min c) 5 min. (scale bar is 100 nm)

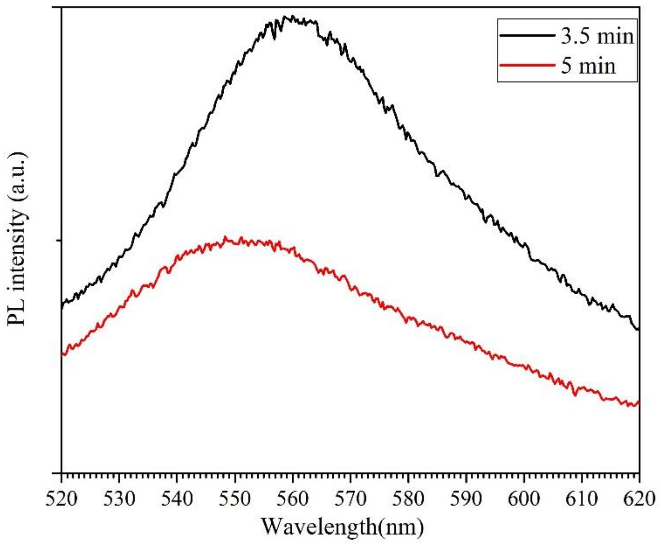

Figure S2: PL spectra of R6G (10^{-5} M) with 405 nm excitation laser for different MACE etching times of Ag-dendrites, 3.5 and 5 min respectively.

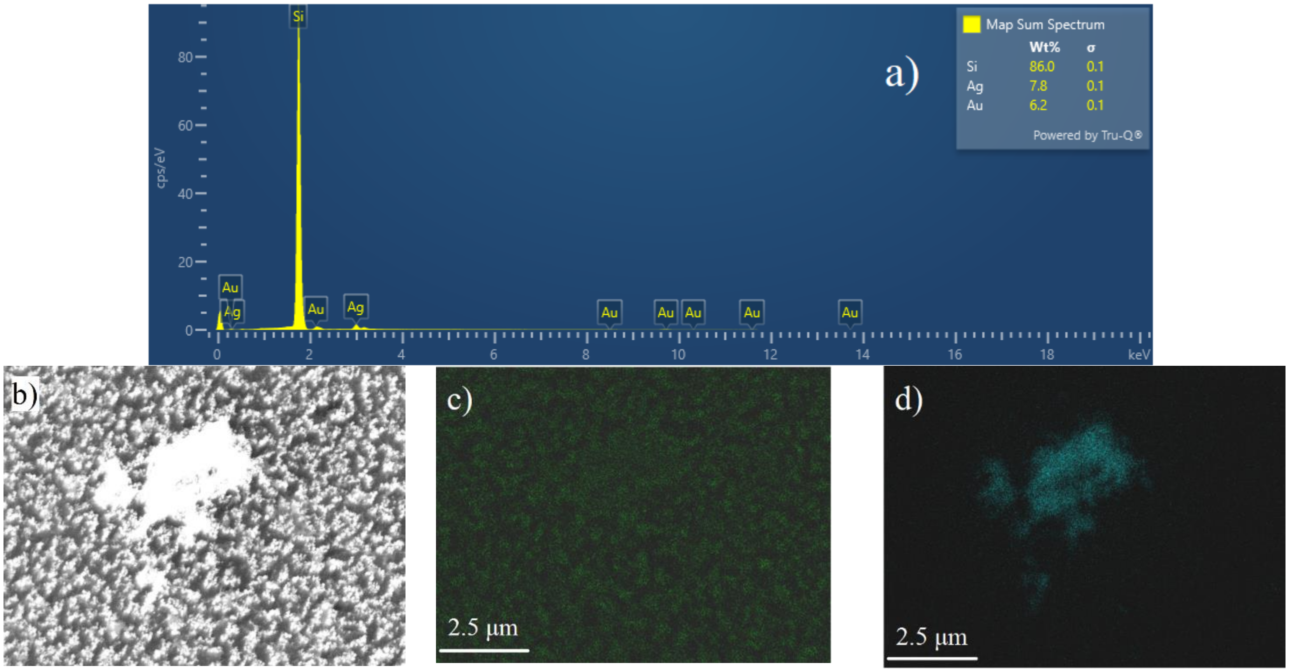

Figure S3. SEM and EDS results of Ag-dendrites/Au substrates at voltage of 20 kV. a) EDS analysis b) SEM image c) EDS map showing detection of Ag d) EDS map showing detection of Au.

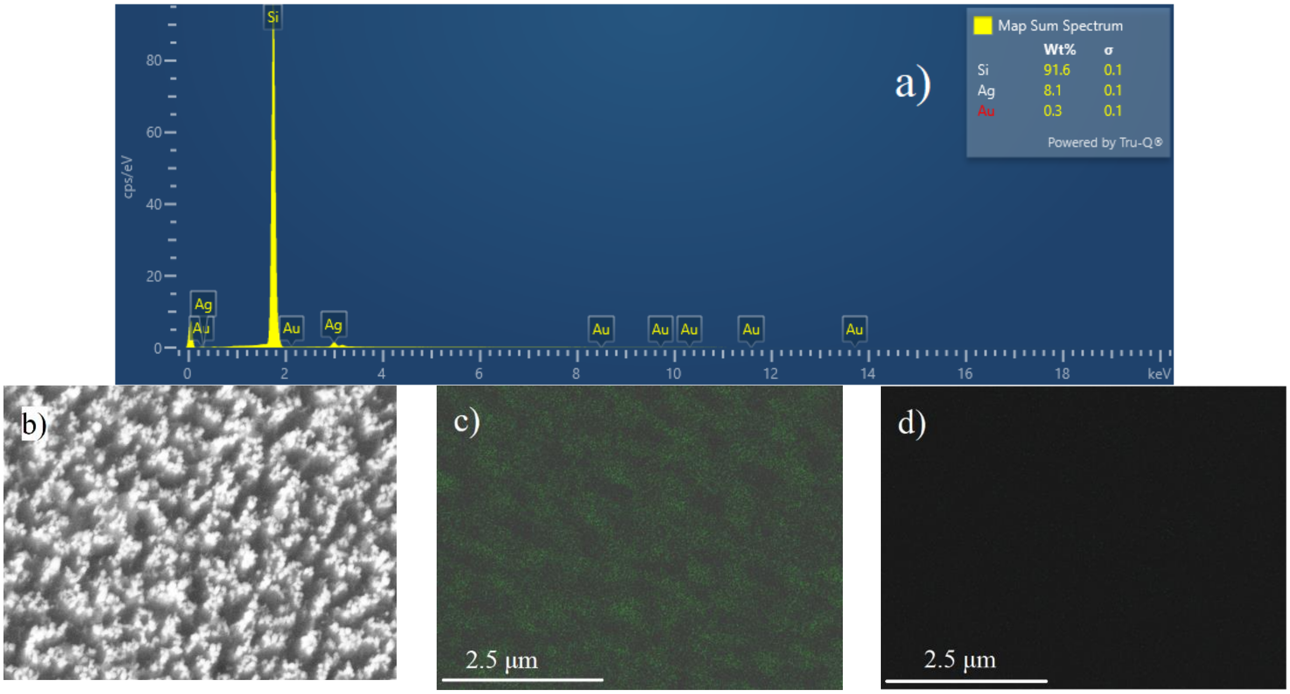

Figure S4. SEM and EDS results of Ag-dendrites substrates at voltage of 20 kV. a) EDS analysis b) SEM image c) EDS map showing detection of Ag d) EDS map showing no detection of Au.

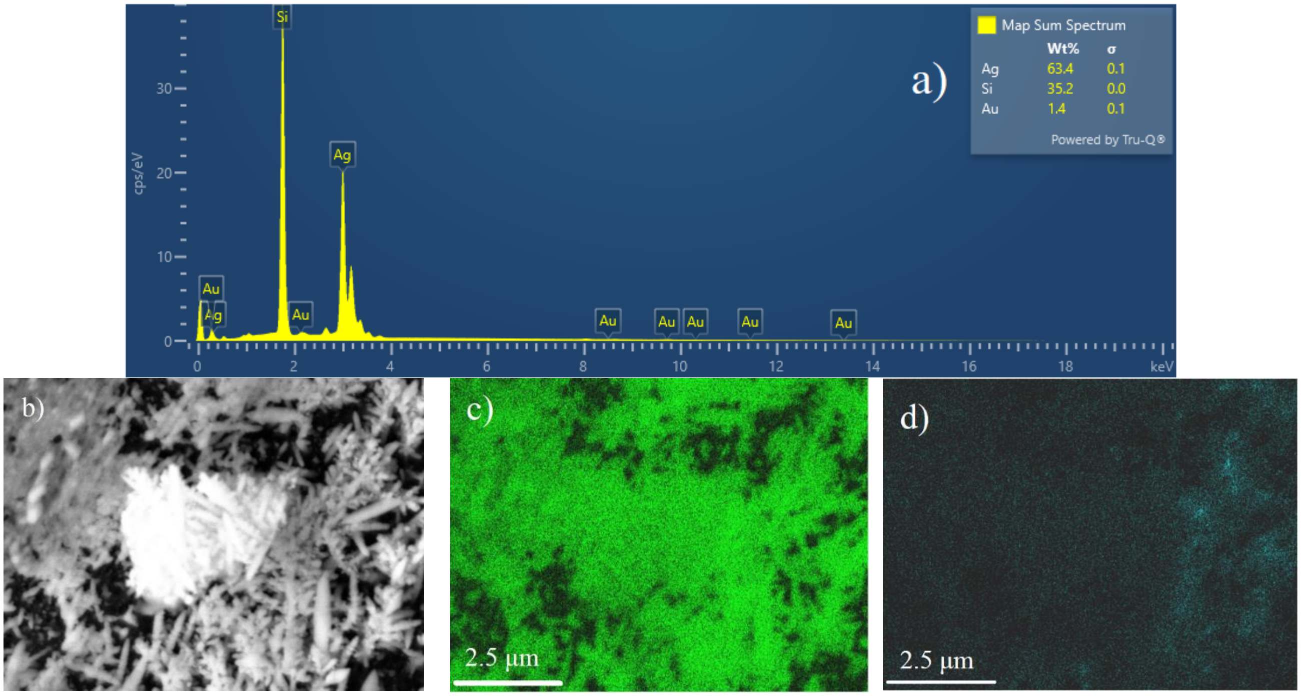

Figure S5. SEM and EDS results of Ag-aggregates/Au substrates at voltage of 20 kV. a) EDS analysis b) SEM image c) EDS map showing detection of Ag d) EDS map showing detection of Au.

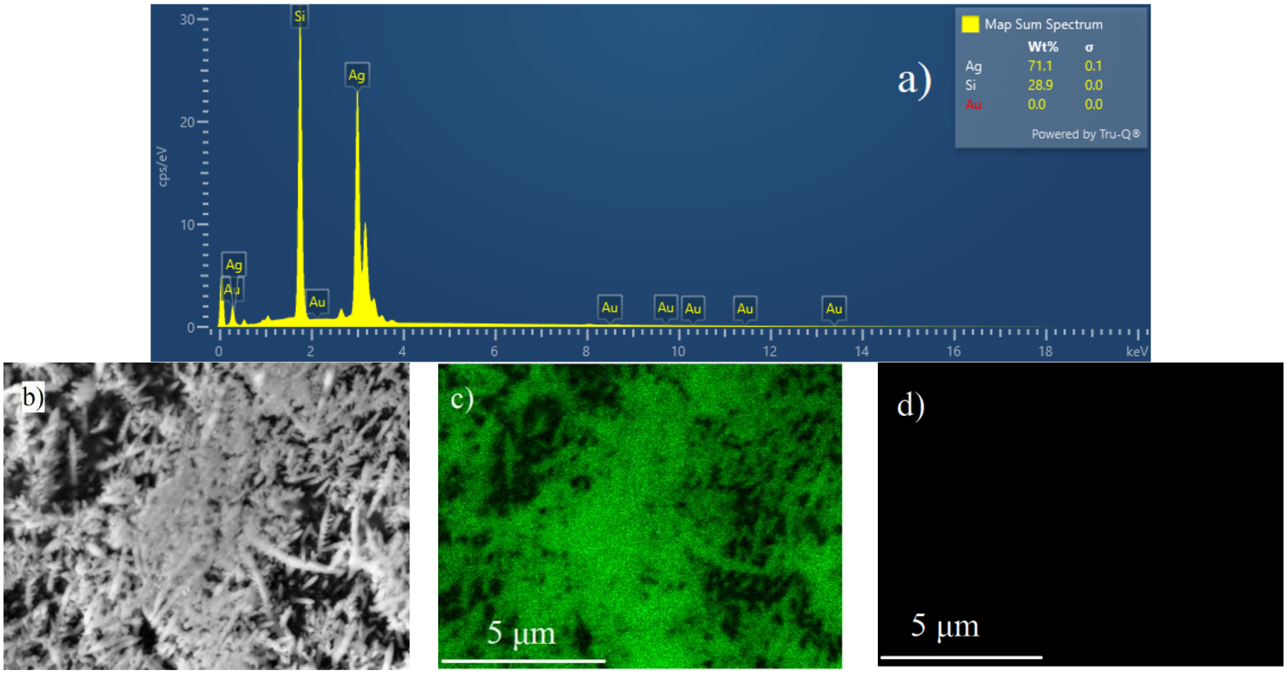

Figure S6. SEM and EDS results of Ag-aggregates substrates at voltage of 20 kV. a) EDS analysis b) SEM image c) EDS map showing detection of Ag d) EDS map showing no detection of Au.

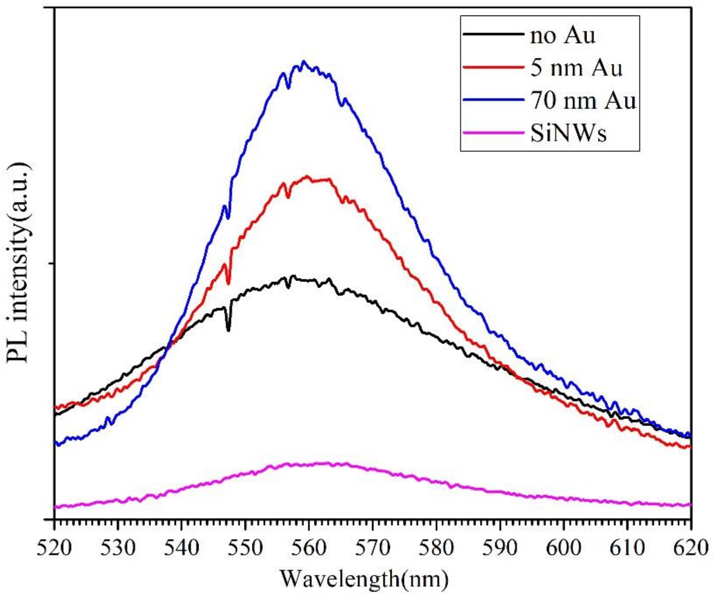

Figure S7: PL spectra of R6G (10^{-5} M) on Aggregates for two different diameters of Au nanoparticles with 405 nm excitation laser in comparison to Au-free Ag-aggregates and SiNWs.

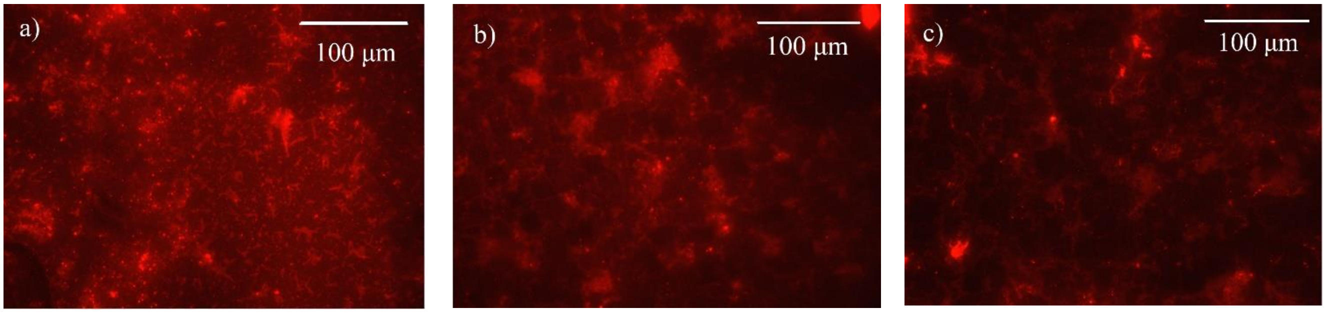

Figure S8. Fluorescence microscope images of Ag-Dendrites/Au with various concentrations of R6G obtained with a TexasRed dichroic filter. a) 10^{-10} M b) 10^{-11} M c) 10^{-12} M.

References

[1]: Phuong, N.T.T.; Nguyen, T.-A.; Huong, V.T.; Tho, L.H.; Anh, D.T.; Ta, H.K.T.; Huy, T.H.; Trinh, K.T.L.; Tran, N.H.T. Sensors for Detection of the Synthetic Dye Rhodamine in Environmental Monitoring Based on SERS. Micromachines 13, 1840 (2022). .

[2]: Pham, T.B., Hoang, T.H.C., Pham, V.H. et al. Detection of Permethrin pesticide using silver nano-dendrites SERS on optical fibre fabricated by laser-assisted photochemical method. Sci Rep 9, 12590 (2019).

Acknowledgements

The authors would like to acknowledge Aggeliki Papavasiliou, researcher in Materials and Membranes for Environmental Separations Laboratory of Institute of Nanoscience and Nanotechnology (INN)- National Center for Scientific Research "Demokritos" for her crucial assistance on the SEM/EDS measurements.
